# Online Educational Intervention on Research Protocol Competencies in Medical Residents: A Quasi-experimental Study

**DOI:** 10.7759/cureus.83505

**Published:** 2025-05-05

**Authors:** Martín Segura-Chico, Nora Delia Nava-Obregrón, Zaida Guadalupe Melgoza-Pelcastre, Emmanuel Alejandro Barrón-Pérez, Brian González-Pérez, Iris Alejandra Benítez-Trejo, Blanca Estela Robles-del Ángel

**Affiliations:** 1 Research, Mexican Institute of Social Security (IMSS), Victoria, MEX; 2 Educational Research and Faculty Training Center, Mexican Institute of Social Security (IMSS), Monterrey, MEX; 3 Educational Research and Faculty Training Center, Mexican Institute of Social Security (IMSS), Victoria, MEX; 4 Planning, Mexican Institute of Social Security (IMSS), Victoria, MEX; 5 Family Medicine, Family Medicine Unit Number 38, Mexican Institute of Social Security (IMSS), Tampico, MEX; 6 Education and Research, General Hospital Number 1, Mexican Institute of Social Security (IMSS), Victoria, MEX; 7 Education and Research, General Hospital Number 6, Mexican Institute of Social Security (IMSS), Madero, MEX

**Keywords:** educational intervention, medical education, online education, protocol design, research training

## Abstract

Introduction

Competence in research methodology and protocol development is essential for medical residents to integrate evidence-based medicine into clinical practice. Online Educational Interventions (OEIs) have been implemented as a structured approach to enhance these skills. This study assesses the impact of an OEI on research methodology knowledge and protocol development among first-year medical residents.

Methods

A quasi-experimental, prospective study was conducted from June 2022 to February 2023 at the Family Medicine Unit No. 1 of the Mexican Social Security Institute (IMSS) in Victoria, Mexico. First-year residents from multiple specialties participated in a structured OEI designed to strengthen competencies in research methodology. Pre- and post-intervention assessments were carried out using a custom-designed questionnaire validated through expert review. Data were analyzed using Wilcoxon signed-rank and paired Student’s t-tests, with statistical significance set at p < 0.05.

Results

A total of 101 medical residents were enrolled in the study, of whom 65 completed the intervention. Among the completers, 39 (60.0%) were female residents, and 26 (40.0%) were male residents. Family Medicine was the most represented specialty, comprising 29 participants (44.6%). Post-intervention assessments demonstrated statistically significant improvements across all evaluated domains (p < 0.05), with the most pronounced gains observed in data collection procedures, participant selection, and study design. The assessment tool showed high internal consistency (Cronbach’s alpha = 0.84).

Conclusions

Structured OEIs significantly improve research methodology knowledge and protocol development skills in medical residents. These findings support their integration into residency training programs to strengthen research capacity and advance evidence-based practice.

## Introduction

The ability to develop research protocols enables medical residents to systematically approach clinical problems using evidence-based methodologies, fostering methodological rigor and contributing to improved decision-making in patient care. The COVID-19 pandemic significantly disrupted medical education in Mexico, particularly in hospitals training medical-surgical residents, where the suspension of in-person activities and reduced clinical exposure highlighted the urgent need for innovative solutions to sustain and improve research training [1,2]. In response, some hospital-based educational programs implemented Online Educational Interventions (OEIs), providing a flexible and accessible platform that ensured continuity in training while helping residents manage demanding schedules and strengthening their critical appraisal skills, methodological rigor, and capacity to conduct original research [3].

These interventions facilitated the development of essential competencies, such as designing clinical protocols, while reinforcing the integration of evidence-based medicine into residency programs. Driven by the challenges of the pandemic, this shift proved crucial in sustaining and strengthening research training, modernizing educational approaches, and fostering a culture of innovation and methodological rigor [4]. By equipping residents with the tools to generate high-quality research, OEIs have become a pivotal mechanism for advancing evidence-based practice and improving training outcomes [5].

This study aimed to evaluate the effectiveness of a structured OEI in enhancing research protocol development competencies among first-year medical residents. Specifically, we evaluated improvements in methodological design, participant recruitment, ethical considerations, and data collection procedures through pre- and post-intervention assessments. The study employed a single-group, pre-post, quasi-experimental design, where the OEI served as the independent variable and domain-specific competency scores as the dependent outcomes.

## Materials and methods

A quasi-experimental prospective study was conducted from June 2022 to February 2023, enrolling first-year medical residents from clinical and surgical care departments at Family Medicine Unit No. 1 of the Mexican Social Security Institute (IMSS) in Victoria, Mexico. Eligible participants were first-year residents who voluntarily enrolled in the online seminar hosted on the Moodle platform (version 4.5.1) (Moodle Pty Ltd, Perth, Australia) and provided signed informed consent. Exclusion criteria included lack of internet access, failure to complete seminar activities, withdrawal of consent at any point during the intervention, or incomplete responses to the assessment instrument. Additionally, residents who discontinued their residency program for any reason during the study period were excluded from the final analysis. The study protocol was approved by the Institutional Review Board, IMSS (approval number R-2022-2804-043).

Educational intervention and structured implementation

The educational intervention consisted of an eight-week structured seminar titled “Research Methodology and Protocol Development for Medical Residents”, delivered via the Moodle platform (version 4.5.1). The program was designed to strengthen methodological competencies through a progressive, task-based format. Each week included a 45-minute synchronous session focused on case discussions or theoretical reinforcement, accompanied by approximately five hours of asynchronous activities such as guided readings, literature reviews, protocol drafting, and interactive assignments. Participants progressed through eight sequential modules, each aligned with a core domain of protocol development (ranging from research objectives and study design to ethical approval and budgeting) culminating in the completion of a full research protocol. All materials were accessible online and monitored through Moodle’s progress-tracking system to ensure engagement.

To evaluate the impact of the intervention, participants completed a pre- and post-intervention assessment using a custom-designed questionnaire. The instrument underwent expert validation by three clinical research specialists. Based on their feedback, minor revisions were implemented to enhance clarity and relevance. The assessment covered core domains such as study design, ethical considerations, literature integration, and statistical planning. Only residents who completed all seminar activities were included in the final analysis. A detailed summary of the weekly session structure is presented in Table 1.

**Table 1:** Session outline of the OEI OEI: Online Educational Intervention

Research protocol development components	Competency goal
1. Strategic Planning and Budgeting	Assessing project feasibility through resource identification, timeline mapping, and logistical planning.
2. Ethical and Bioethical Considerations	Applying ethical standards, regulatory compliance, and risk-benefit analysis.
3. Data Collection Instruments	Creating valid, reliable, and context-appropriate instruments for data acquisition.
4. Methodological Design	Selecting appropriate study designs, sampling techniques, and calculating sample size.
5. Theoretical Framework and Literature Review	Constructing conceptual frameworks and managing bibliographic databases.
6. Establishing Research Objectives	Formulating research questions and objectives with methodological alignment.
7. Developing a Research Protocol	Synthesizing all core elements into a coherent, methodological research protocol.
8. Foundations of Scientific Research	Differentiating research paradigms and their relevance in clinical investigation.

Statistical analyses were conducted using IBM SPSS Statistics for Windows, Version 25.0 (IBM Corp., Armonk, NY, USA). Descriptive statistics summarized sociodemographic data. Normality was assessed using the Kolmogorov-Smirnov test with Lilliefors correction. When deviations from normality were observed, non-parametric methods (Wilcoxon signed-rank test) were applied; otherwise, parametric methods (paired Student’s t-test) were used. The significance level of p < 0.05 was established. A post hoc power analysis using G*Power (version 3.1.9.7) (Heinrich-Heine-Universität Düsseldorf, Düsseldorf, Germany) confirmed that the final sample size (n = 65) provided >80% power to detect medium to large effect sizes.

## Results

A total of 101 first-year medical residents enrolled in the OEI, representing the full cohort from clinical and surgical departments at the Family Medicine Unit No. 1 of the IMSS during the 2022-2023 academic cycle. Participant withdrawal was primarily attributed to academic overload, scheduling conflicts with clinical duties, and limited availability during the asynchronous components. Of these, 36 residents (35.6%) were excluded due to non-completion of course activities or failure to meet final evaluation requirements. The final study sample consisted of 65 residents, of whom 39 (60.0%) were female participants and 26 (40.0%) were male participants. The mean age was 28 years, with a range from 24 to 46 years. The overall completion rate was 64.4%. The intervention was offered exclusively at this institution, and no external residents were eligible for inclusion.

The distribution of participants across residency programs showed a predominance of Family Medicine residents, accounting for 29 (44.6%) of the total sample, followed by Pediatrics with eight (12.3%) and Gynecology and Obstetrics with six (9.2%) participants. Additionally, 11 residents (16.9%) reported having received mandatory research training prior to the OEI, while eight (12.3%) had previously completed mandatory statistics training. These findings provide a baseline profile of participants' prior exposure to research-related education before the intervention (Table 2).

**Table 2:** Participants’ demographics, perceptions on their knowledge and confidence related to OEI OEI: Online Educational Intervention

Parents characteristics	Pre-course N = 101 (%)	Post-course N= 65 (%)
Sex, n (%)		
-Female	59 (58.4)	39 (60.0)
-Male	42 (41.6)	26 (40.0)
Age, mean (Range)	28 (24-46)	
Residency program, n (%)		
Anesthesiology	10 (9.9)	4 (6.2)
Gynecology and Obstetrics	6 (5.9)	6 (9.2)
General Surgery	4 (4.0)	4 (6.2)
Occupational Medicine	6 (5.9)	5 (7.7)
Family Medicine	35 (34.7)	29 (44.6)
Traumatology and Orthopedics	8 (7.9)	5 (7.7)
Pediatrics	11 (10.9)	8 (12.3)
Emergency Medicine	21 (20.9)	4 (6.2)
Mandatory research training in their institution/college, n (%)		
-Yes	11 (10.9)	11 (16.9)
-No	90 (89.1)	54 (83.1)
Mandatory statistics training in their institution/college, n (%)		
-Yes	8 (7.9)	8 (12.3)
-No	93 (92.1)	57 (87.7)

**Figure 1:**
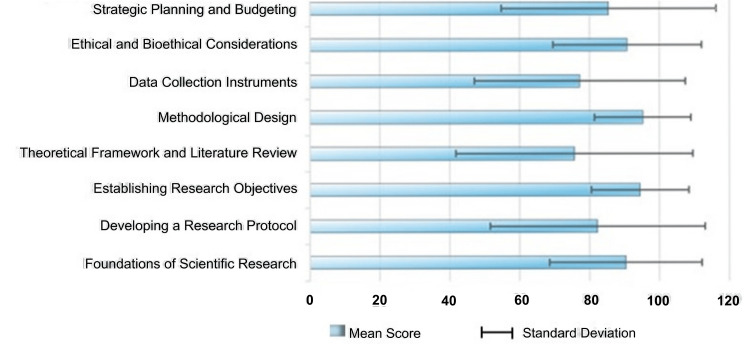
Participants’ mean scores by session component of OEI OEI: Online Educational Intervention

The assessment of participants' performance across the eight components of the OEI revealed heterogeneous mean scores, reflecting varying levels of comprehension and applied proficiency in each domain. The highest mean was observed in "methodological design" (95.2, SD 13.79), followed closely by "establishing research objectives" (94.46, SD 14.00) (Figure 1). Similarly, participants demonstrated strong performance in "ethical and bioethical considerations" (90.77, SD 21.27) and "foundations of scientific research" (90.34, SD 21.85), indicating a solid grasp of core methodological and ethical principles.

Conversely, the lowest mean score was recorded in "theoretical framework and literature review" (75.66, SD 33.86), suggesting that conceptual integration and academic referencing may pose greater challenges for first-year residents. A relatively lower performance was also observed in "data collection instruments" (77.2, SD 30.16). Moderate scores were obtained in "developing a research protocol" (82.31, SD 30.77) and "strategic planning and budgeting" (85.46, SD 30.71), indicating acceptable but improvable proficiency in synthesizing and operationalizing protocol components (Figure 1).

The comparative analysis of pre- and post-course assessments demonstrated statistically significant improvements across all evaluated domains (p < 0.05). The most substantial gain was observed in "data collection procedures", with scores increasing from 4.52 (SD 2.92) to 6.86 (SD 1.17) (p = 0.000) (Table 3). Similarly, "participant selection and recruitment" improved from 4.15 (SD 3.00) to 6.70 (SD 1.33) (p = 0.000), and "study design and methodology" rose from 4.89 (SD 3.06) to 6.55 (SD 1.23) (p = 0.000), indicating a strengthened understanding of core methodological concepts.

**Table 3:** Comparative analysis of pre- and post-course results of OEI OEI: Online Educational Intervention, Asymp. Sig.: asymptotic significance

	Pre-course			Post-course			
	Mean (SD)	Kurtosis	Asymp. Sig.	Mean (SD)	Kurtosis	Asymp. Sig.	P
Project title and identification	5.41 (2.82)	-1.31	0.33	7.23	-1.13	-0.19	0.000
Background and literature review	4.98 (3.06)	-1.48	0.40	6.61	-1.09	-0.17	0.000
Research objectives	4.58 (3.07)	-1.21	0.68	6.30	-1.24	0.14	0.001
Study design and methodology	4.89 (3.06)	-1.33	0.54	6.55	-1.23	-0.15	0.000
Participant selection and recruitment	4.15 (3.00)	-0.60	1.03	6.70	-1.33	-0.21	0.000
Data collection procedures	4.52 (2.92)	-0.76	0.80	6.86	-1.17	-0.34	0.000
Ethical considerations	4.98 (3.10)	-1.25	0.59	6.86	-1.06	-0.33	0.000
Data management and statistical analysis	4.89 (2.98)	-1.04	0.60	6.18	-1.11	-0.13	0.010
Potential risk and benefits	4.76 (2.73)	-0.82	0.66	6.24	-1.09	-0.08	0.001
Study timeline and budget	4.76 (2.73)	-1.13	0.66	6.09	-0.94	0.75	0.003

Notable improvements were also recorded in "project title and identification" (from 5.41 to 7.23, p = 0.000), "ethical considerations" (from 4.98 to 6.86, p = 0.000), and "background and literature review" (from 4.98 to 6.61, p = 0.000), with mean differences ranging from 1.63 to 1.88 points. The smallest gain was observed in "study timeline and budget", increasing from 4.76 (SD 2.73) to 6.09 (SD 0.94) (p = 0.003), suggesting this topic may require further instructional reinforcement.

Kurtosis values remained within acceptable ranges, indicating no significant departure from normality. Slight deviations in asymptotic significance further supported the use of non-parametric methods for the analysis. Overall, these results confirm the effectiveness of the OEI in enhancing key competencies for research protocol development among participating residents (Table 3).

In summary, the statistically significant improvements observed across all domains, particularly in methodological design, participant selection, and data collection procedures highlight the effectiveness of a short-format, assignment-based online course delivered early in residency. These results suggest that the OEI successfully addressed baseline deficiencies in research training among first-year residents.

## Discussion

This study demonstrates that early, structured online educational interventions can effectively strengthen research methodology competencies among first-year medical residents. By engaging participants in progressive, assignment-based learning, the OEI contributed to improved proficiency in designing protocols, applying ethical principles, and planning data collection (skills that are often underdeveloped in the initial stages of postgraduate training). These findings align with previous reports emphasizing the importance of early research training in medical residency programs [4,5], and support growing evidence that digital platforms, when properly structured, can bridge gaps in research education, particularly in resource-constrained academic environments.

Beyond technical knowledge, the structured design of the intervention promoted analytical thinking and academic rigor, both essential for generating locally relevant evidence in clinical settings. In resource-constrained environments, where formal research training is often limited, early exposure to practical research exercises, such as assignment-based protocol construction may help bridge this gap and foster long-term engagement with scientific inquiry [6].

Assignment-based research training has been shown to enhance critical thinking, improve literature appraisal, and strengthen methodological precision [7,8]. Kofi et al. demonstrated that structured, iterative research assignments significantly improve residents’ ability to evaluate and apply scientific evidence [9]. Other studies have likewise reported that structured interventions enhance understanding of statistics, ethics, and protocol development-critical steps in both clinical and translational research [10]. The OEI’s online format, delivered asynchronously with structured assignments and guided reading, provided a flexible framework for research training within the demands of residency. While the course lacked real-time mentoring, its scaffolded design allowed residents to engage with content at their own pace and progressively build the core components of a research protocol, resulting in measurable improvements in competency [11].

Solbach-Sabbach et al. further supports the effectiveness of blended learning through an inverted classroom model for family medicine residents, in which theoretical content was delivered asynchronously, while synchronous sessions focused on case-based learning, interactive discussions, and peer mentoring [12]. The transition from traditional to online research training has presented challenges, particularly in resource-limited settings, where barriers such as limited digital infrastructure, insufficient faculty training for online instruction, and reduced opportunities for interactive learning persist. Fauzia et al. underscores the need for adaptive strategies that enhance accessibility, engagement, and equitable participation in online research training programs [13].

The impact of the OEI can also be contextualized through comparison with standardized assessment tools. Argimon-Pallàs et al. reported significant knowledge gains using the Fresno Test [14]. Similarly, our study demonstrated improvements in core domains, including study design, ethics, recruitment, and data analysis. While the Fresno Test assesses broad evidence-based practice skills, our evaluation focused specifically on protocol development, underscoring the importance of aligning assessment tools with educational goals.

The strengths of this study include a structured educational design tailored to protocol development competencies, expert-validated evaluation instruments, and statistically powered analyses. However, the absence of a control group inherent to the quasi-experimental, single-group design limits the ability to establish causal relationships between the intervention and the observed improvements. The study was conducted at a single institution, which may restrict the generalizability of findings. Furthermore, the long-term retention of research skills was not assessed, highlighting the need for follow-up studies with extended evaluation periods.

## Conclusions

This quasi-experimental study provides evidence that structured OEIs are a valuable pedagogical approach to improving core research competencies among first-year medical residents. By offering a task-based, asynchronous format tailored to the demands of residency, the intervention facilitated the acquisition of critical skills such as study design, ethical analysis, and protocol construction, which are elements often underemphasized in early postgraduate training. These findings emphasize the importance of integrating research-oriented education early in medical training, not only to enhance methodological literacy but also to promote evidence-based clinical reasoning and sustained engagement with scientific inquiry. Incorporating OEIs into residency curricula may serve as a scalable, cost-effective strategy to reinforce academic rigor, bridge gaps in formal research instruction, and foster long-term engagement with scientific investigation. Further multi-center, longitudinal studies are needed to assess the durability of these competencies and their translation into academic productivity and clinical innovation.
